# Floral induction in response to environmental cues: extending a century of progress

**DOI:** 10.1007/s00425-026-05018-7

**Published:** 2026-05-11

**Authors:** George Coupland

**Affiliations:** Department of Plant Developmental Biology, Max Planck Institute for Plant Breeding Research, Cologne, Germany

**Keywords:** Florigen, Flowering regulators, Quantitative models, Transcription factors, Vernalization

## Abstract

**Main conclusion:**

Great progress has been made in understanding the mechanisms of floral induction based on physiology and molecular genetics, whereas recent research suggests that future developments will come from protein biochemistry, genomics, imaging, and mathematical modeling.

**Abstract:**

During the twentieth century, plant physiologists showed how the developmental transition from vegetative growth to flowering is controlled by environmental cues, such as day length and temperature. They defined a number of interesting questions such as the identity of the signal induced in response to day length in leaves that is translocated to the shoot meristem to induce flowering, and the mechanism by which plants sense and remember exposure to winter temperatures. Application of molecular-genetics approaches from the 1990s identified regulatory proteins and small RNAs that control these responses, first in Arabidopsis and later in several crops. These advances identified a series of new questions. In this Perspective article, I highlight some of these issues and select some recent papers that show how protein biochemistry, genomics, imaging, and mathematical modeling address these questions, and will in turn improve our understanding of the mechanisms of floral induction.

## Introduction

**Fig. 1:**
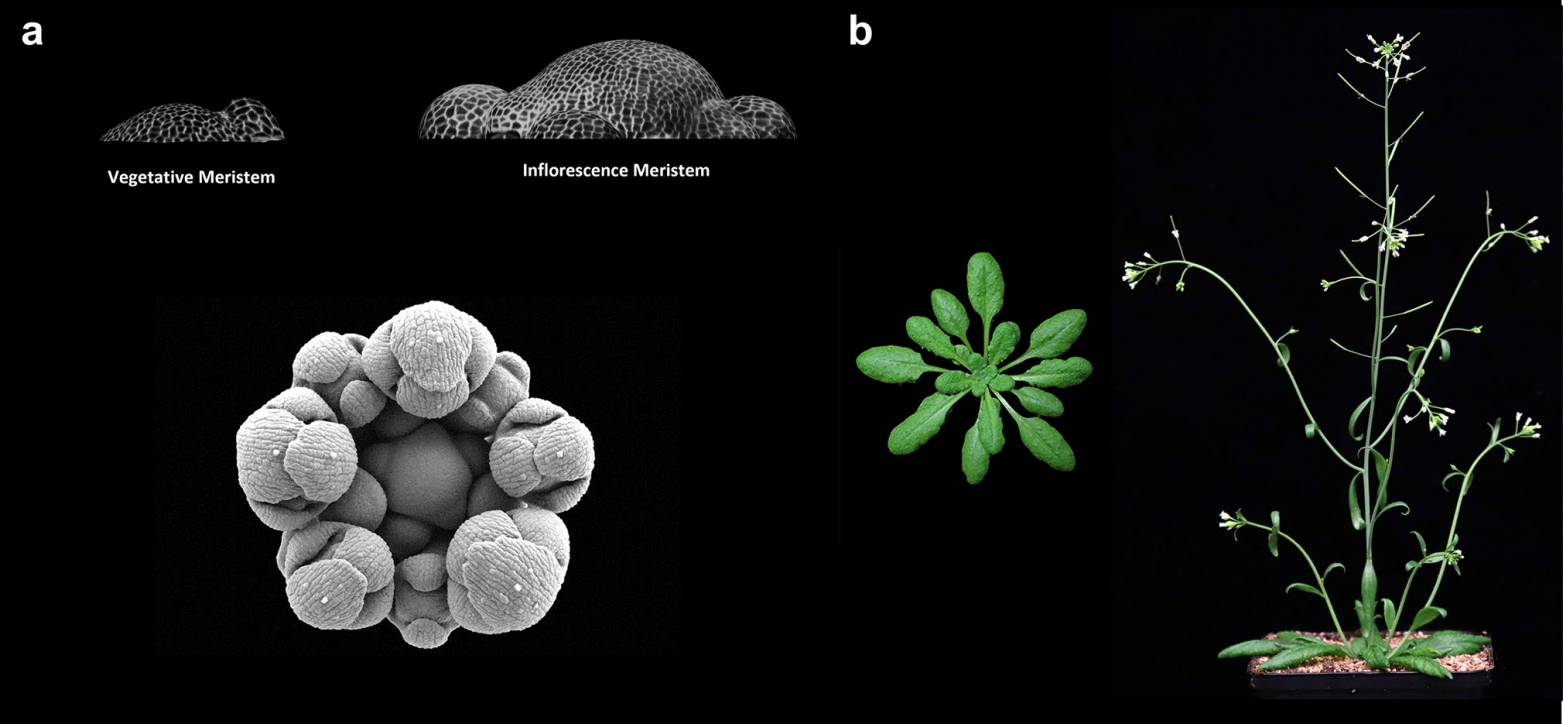
Floral transition in Arabidopsis and related Brassicaceae. **a** Development of the shoot apex. Top left, vegetative shoot apical meristem of Arabidopsis with leaf primordia. Top right, larger inflorescence meristem with floral primordia. Bottom, older inflorescence meristem with developing floral buds in a spiral pattern, here illustrated with an apex of *Arabis alpina*. **b** Arabidopsis plants before and after floral transition. Left, vegetative rosette forming leaves in a spiral pattern. Right, Flowering plant. The primary shoot has elongated to form the inflorescence. The first nodes on the inflorescence form branches, paraclades, in the axils of the cauline leaves. At later nodes, solitary flowers are formed

This Perspective on floral induction is part of the *Planta* Centennial Collection. The first issue of *Planta* was published in 1925 with the aim of providing a forum for advances in plant science. It is no coincidence that the American Society of Plant Biologists (ASPB) was founded the year before, because great advances in understanding plant science were made in the 1920s. One of these was the discovery of photoperiodism (Garner and Allard 1920). The insight that plants could measure day length and use this information to control the time of flowering led to the discovery of phytochrome photoreceptors and to the formulation of the florigen hypothesis that systemic signals formed in the leaf were translocated to the apex and trigger floral development at the shoot meristem. The problems of sensitivity to day length, the nature of florigen, and the related question of how exposure to cold during winter (vernalization) promotes flowering in spring were intensively studied by physiologists for over 70 years. In his review of 1971, Lloyd Evans mentions that 1200 papers had been published on flowering since the previous review in the series 6 years earlier (Evans 1971). A magisterial review by Anton Lang covered in great depth the physiology of day length and vernalization responses and their history (Lang 1965). In the bibliographies of these and other reviews, papers in *Planta* were strongly represented. Several of the questions set by these physiological studies were finally (partially) solved by identification of mutations or natural genetic variation that altered the response of flowering time to day length or vernalization, and by the development of methods to isolate the underlying genes. Initially, this approach was possible in greatest depth in Arabidopsis (Fig. 1), but impressive advances were later made in crops, including rice, tomato, maize, wheat, barley, and the legumes pea and soybean. These studies identified the major genes and proteins that regulate floral induction and, in some cases, placed them in gene regulatory networks with some understanding of when and where they act during floral transition (Andrés and Coupland 2012). Advances in microscopy have also provided us with detailed descriptions of the changes that occur at the shoot apical meristem (SAM) on flowering and during the early stages of floral development, a process called evocation by the earlier physiologists. The importance of the time of flowering in ensuring fitness in natural populations and in maintaining or increasing yields in agriculture mean that a deep understanding of the process is as important as ever (Fulgione et al. 2022; Park et al. 2014). But looking forward, it is important to consider what the remaining questions are and how we can answer them. Below I describe some pointers from recent research, with an emphasis on work in Arabidopsis, and highlight some of the open questions.

## Biochemistry and protein structures

Advances in protein biochemistry and structural biology have deepened understanding of other areas of plant biology, such as hormone signaling and plant disease resistance. Many of the critical regulators of flowering identified through genetics have been shown to control transcription of specific target genes or to be indirect transcriptional regulators that influence histone modifications. The application of cryo-EM to plant transcriptional regulatory complexes involved in flowering holds promise for increasing our understanding of the specificity of these complexes and of how distinct flowering pathways are integrated at common target genes. The potential of this approach is illustrated by studies on the interaction of the UNUSUAL FLORAL ORGANS (UFO) and LEAFY (LFY) proteins, which were initially identified from mutations that impair flower development. These proteins had been shown genetically to cooperate to regulate gene expression during floral development, but the mechanism was difficult to define, because LFY is a DNA-binding transcription factor, whereas UFO was proposed to be a ubiquitin ligase on the basis of its homology to F-box proteins (Lee et al. 1997). However, biochemical analysis of DNA-binding specificity combined with cryo-EM showed that the UFO–LFY complex binds to different target sites than LFY alone (Rieu et al. 2023). The UFO–LFY-binding sites contain a motif where UFO unexpectedly interacts with DNA as well as a weak or non-canonical LFY-binding site. The combination of the interaction between the proteins and the spacing of the UFO and LFY recognition sites confers synergy between UFO and LFY enabling LFY–UFO to recognize different sites and activate different sets of target genes than LFY alone. Similar approaches with other combinations of transcription factors that exhibit genetic interactions suggesting they act synergistically will deepen our understanding of the flowering process. A long-standing problem of this type is how combinations of MADS-box transcription factors form tetrameric complexes to regulate different sets of target genes. These complexes can act as promoters or repressors of flowering time, or as regulators of floral development. More than 100 genes encoding MADS-box transcription factors are present in the Arabidopsis genome, several of which promote or repress various steps in the flowering process, but how the specificity of their protein interactions and the selection of their target sites are determined remains poorly understood. Nevertheless, structure–function analyses are beginning to define the mechanisms that confer MADS-box specificity (Lai et al. 2021). Related issues arise in understanding the interactions between the FD and SQUAMOSA PROMOTER BINDING PROTEIN-LIKE (SPL) transcription factors. FD is a bZIP transcription factor that acts in the Arabidopsis photoperiodic flowering pathway to promote flowering under long days (LDs), whereas SPLs represent a family of plant-specific transcription factors that promote flowering mainly under short days (SDs). FD and SPLs bind to different DNA motifs but share many target genes and have been proposed to physically interact to regulate gene expression (Jung et al. 2016). A deeper understanding of the biochemistry of these interactions may reveal unexpected synergies between flowering pathways that have so far been mainly considered to function independently.

The florigen activation complex (FAC) poses fascinating biochemical questions that would have pleased the pioneering physiologists. This complex contains FLOWERING LOCUS T (FT), a small protein with similarity to phosphatidylethanolamine-binding proteins that promotes flowering and is expressed in the vasculature of leaves in response to inductive photoperiods. This small protein moves from the leaves to the SAM where it promotes flowering, and is now often referred to as florigen protein (Gao et al. 2026). At the shoot apex, two copies of FT form a transcriptional complex with two copies each of the FD bZIP transcription factor and 14-3-3 protein. FD is proposed to recruit the FAC to specific G-box motifs in target genes. Partial structures for this complex were initially described using rice proteins (Taoka et al. 2011). More recently, modeling of the structure of the Arabidopsis FAC predicted that the previously unstudied C-terminus of FT interacts with DNA, and this was supported by mutagenesis and functional analysis in vivo and in vitro (Gao et al. 2025). Structural data for the full FAC bound to DNA, together with further in vivo and in vitro biochemical studies, will help determine whether FT influences the strength of DNA binding of the FAC and/or its specificity. Similarly, the biochemical role of FD within the FAC is not fully understood. It dimerizes through the leucine zipper domain and binds DNA as a dimer via the adjacent basic domain, while a phosphorylated residue (T282) close to the carboxy terminus of the protein is recognized by the 14-3-3, which also interacts with FT. However, the basic and leucine zipper domains as well as the 14-3-3 binding site are all located in the carboxy-terminal part of FD, and over 200 amino acids at the largely disordered N-terminus have not yet been ascribed a function. However, the N-terminus of FD shares short segments of homology with other Group A bZIPs involved in abscisic acid (ABA) signaling for which phosphorylation of the homologous regions is required for transcriptional activation (Martignago et al. 2025). Notably, the N-terminus of FD does not contain the residues phosphorylated in the ABA bZIPs, but the surrounding regions around these residues represent conserved motifs. Therefore, understanding how the long, disordered N-terminus of FD contributes to the activity of the FAC, and whether it contributes to the promotion of transcription by the FAC, is an important question for the future. Another unresolved biochemical problem is how the specificity between FT and other PEBP proteins is conferred. The activity of FT at the shoot apex is modulated by the related protein TFL1. Whereas FT promotes flowering, TFL1 is a repressor of flowering that maintains indeterminacy of the inflorescence meristem by preventing its conversion into a solitary flower. A conserved external loop in FT proteins from diverse species is important for activity of the protein and has strongly diverged in TFL1. Indeed, transfer of segment B from TFL1 to FT is sufficient to convert FT into a floral repressor (Ahn et al. 2006). Moreover, in several species such as sugar beet and soybean, paralogues of FT have evolved independently as floral repressors, and these most strongly diverge from canonical FT proteins in the sequence of segment B. This region of FT is proposed to be required for the FAC to activate transcription of target genes, which is proposed to be impaired in TFL1 and the FT paralogues that modulate FT activity (Ahn et al. 2006). However, the precise biochemical function of segment B remains unknown, and is likely to be key to further testing the contribution of FT and TFL1 to regulating transcription of target genes and their antagonism during flowering.

In the vernalization response, important questions remain, requiring detailed protein biochemistry. In Brassicaceae, vernalization is conferred by the MADS-box transcription factor FLOWERING LOCUS C (FLC), which acts as a repressor of flowering before cold exposure (Michaels and Amasino 1999; Sheldon et al. 1999). However, in the cold, *FLC* transcription is reduced and histone modifications at *FLC* are altered from those associated with active transcription [trimethylation of lysine 36 of histone 3 (H3K36me3) and trimethylation at lysine 4 of histone 3 (H3K4me3)] to those associated with transcriptional repression [trimethylation of lysine 27 on histone 3 (H3K27me3)] (Yang et al. 2014). The FRIGIDA (FRI) protein is required for active transcription of *FLC* prior to cold exposure and indirectly for the deposition of H3K4me3 and H3K36me3 at *FLC*. Recent work showed that in the cold, FRI is rapidly stabilized and forms high-molecular-weight nuclear condensates away from the *FLC* locus (Zhu et al. 2021). Formation of these condensates is proposed to represent an early step in *FLC* transcriptional repression and requires disordered regions within FRI. These condensates contain many other proteins. Describing the protein components of these condensates and how protein interactions within it stabilize FRI leading to the formation of liquid-like particles sequestering FRI from *FLC* will provide insight into how temperature is sensed at the onset of the vernalization response.

## Understanding flowering pathways with cellular resolution

A feature of floral transition is the integration at the SAM of multiple signals that control the precise timing of flowering. These signals originate in different tissues of the plant, including movement of FT from leaves and developmental signals associated with the age of the shoot. The tissue-level control of floral transition was first defined by grafting and later by expressing flowering-time genes from tissue-specific promoters. However, the precise cell types in which flowering pathways and their component proteins function remain generally poorly defined. An exception is the activation of the photoperiodic pathway module CONSTANS (CO)–FT, which, based on confocal microscopy of FT protein fusions to fluorescent proteins and marker proteins specific to vascular cell types, indicates that the FT protein is produced in response to inductive day lengths specifically in the companion cells of the mature phloem (Chen et al. 2018; Gao et al. 2025). However, other aspects of flowering pathway function are not yet understood at the cellular level and this is now tractable using detailed confocal and electron microscopy and single-cell sequencing across the floral transition, as was performed recently in maize (Dong et al. 2026). For example, a second day-length-independent pattern of *FT* transcription was recently identified at the shoot apex, following the movement of FT protein to the apex during the early stages of floral transition. Imaging and fluorescent in situ hybridizations demonstrated that this pattern includes a patch of cells on the adaxial side of the cauline leaf, the boundary between the SAM and the primordium, and the boundary between the floral meristem and the suppressed bract (Gao et al. 2025). The identity of these cells relative to those expressing other boundary genes, how these precise transcriptional patterns are controlled, and whether a regulatory hierarchy exists between FT and other boundary genes remain unclear.

The temporal and spatial regulation of the floral transition at the shoot apex also requires more detailed cellular description. Several developmental processes at the shoot apex are closely associated with floral transition and their precise cellular locations are not well defined. For example, within a day after transferring Arabidopsis plants from SDs to LDs, *FT* transcription occurs in the vasculature of the leaves, and during the following 4–5 days, the SAM increases in size and alters in shape, the rib region and upper stem begin to elongate in preparation for bolting of the inflorescence stem, leaf development is suppressed while axillary meristems in the axils of cauline leaves are activated to form paraclades (inflorescence branches), and newly formed primordia adopt floral identity (Kinoshita et al. 2020). Many questions remain in understanding how the gene regulatory networks that induce flowering are expressed in different cell types at distinct times across the shoot apex to control these different aspects of flowering and inflorescence development. Moreover, single flowering genes may regulate several of these processes and how their specificity is conferred is unclear. Examples include *FRUITFULL*, which encodes an MADS-box transcription factor that has roles in promoting floral transition, stem bolting, cauline leaf suppression and floral meristem identity, as well as fruit growth (Ferrandiz et al. 2000; Chen et al. 2025). *FUL* transcription is induced during floral induction and is broadly expressed throughout the shoot apex. It is probably transcribed in different gene regulatory networks in diverse cell types where it forms distinct protein complexes with various other MADS-box transcription factors to contribute to different phenotypes (Chen et al. 2025). Recently, single-nucleus sequencing linked FUL to the regulation of cell division via activation of *KRP2*, which encodes a cell division repressor (Chen et al. 2025). By this means, FUL is proposed to be involved in transitioning cells from division to differentiation during flowering. However, this aspect of FUL function remains to be spatially and temporally integrated into its multiple roles during floral transition. Similarly, the APETALA2 (AP2) transcription factor is widely expressed in the SAM during vegetative growth and is a repressor of floral transition (Yant et al. 2010). However, it is also required to increase SAM height during floral transition and to increase the number of cauline leaves and inflorescence paraclades that form before floral development (Bertran Garcia de Olalla et al. 2024). The cells at the shoot apex in which AP2 functions to increase SAM height and how these compare with those required to increase paraclade number remains unclear.

The cell biology of how phloem contents are downloaded at the Arabidopsis shoot apex and how this contributes to floral transition is also a pressing issue. Early physiological approaches suggested increased sink strength at the apex played a significant role in floral induction (Bernier 1988). Studying dye unloading at the SAM suggested symplastic downloading and that this varied in efficiency at different stages of flowering (Gisel et al. 2002). Increased sink strength may also explain the higher levels of sucrose and trehalose-6-phosphate (T6P) sugars detected at the Arabidopsis apex during floral transition (Wahl et al. 2013). Understanding phloem downloading will be important to explain how FT protein moves from the end of the mature phloem sieve elements through the protophloem into the rib region and the SAM. Although plasmodesmata proteins have been identified that are required for FT movement from the companion cells into the sieve elements (Liu et al. 2012), we do not know whether the same proteins are involved in movement into the SAM. However, recent work showed that blocking plasmodesmata in the apex impairs FT movement in the SAM (Murata et al. 2025). Studying the cell biology and dynamics of phloem FT downloading at the SAM will likely provide another valuable link between classical physiological concepts of sink strength and modern molecular genetics and imaging.

## Quantification and modeling

The precise timing of floral transition is determined by the quantitative balance between repressors and activators of flowering at the shoot apex. Many of these include proteins that activate or repress transcription, or microRNAs that negatively regulate key transcription factors. The balance between these activators and repressors of flowering is shifted by environmental cues such as day length or vernalization and by the age of the plant. Quantification of key regulatory molecules at the SAM by confocal microscopy can provide a measure of the output of these pathways and allow the dynamics of floral transition to be mathematically modeled. For example, quantification of the AP2 repressor of flowering and of the SUPPRESSOR OF OVEREXPRESSION OF CONSTANS 1 (SOC1) and FUL proteins that promote floral transition led to the formulation of a mathematical model that can recreate the experimentally measured decline in AP2 abundance during floral transition and the timing of initiation of flower development (Rodríguez-Maroto et al. 2025). AP2 levels do not decline linearly but are characteristically reduced rapidly, slowly, and then rapidly again, as the plant proceeds to flowering. This pattern creates a transient stage during which AP2 is expressed at intermediate levels and that delays floral development and extends the floral transition. Notably, this intermediate stage corresponds to, or overlaps with, the time at which cauline leaves and paraclades are formed. Therefore, the dynamics of AP2 reduction and the resulting delay in floral development might determine the number of inflorescence branches formed prior to floral development and thereby influence inflorescence architecture. Accordingly, *ap2* mutants form fewer paraclades, whereas extending AP2 expression in *soc1* mutants increases their number (Bertran Garcia de Olalla et al. 2024). Therefore, the rate with which the transition from expression of repressors of flowering to the expression of activators of floral transition occurs at the SAM during the floral transition can strongly influence inflorescence morphology. Such approaches indicate that genetic control of flowering time and the dynamics of floral transition are intimately involved in regulating inflorescence architecture. Analysis of the transcriptomes of staged meristems from five Solanaceae species also indicated that the rate of SAM maturation underlies inflorescence architecture (Lemmon et al. 2016). These results suggest that quantitative models of floral transition may have applications in breeding by predicting responses to environmental cues, as well as in generating rational strategies for manipulating inflorescence or spike development in crops, with implications for yield.

## Concluding remarks

Although the pioneers of flowering physiology did not for the most part use a genetic approach, they would surely appreciate the progress that has been made and generally recognize how the genes and proteins identified by molecular genetics relate to the physiological concepts that they defined. However, they might find some aspects surprising and have expected more progress on others. Generally, physiologists used a broader range of model species, because they were not constrained by the practicalities of molecular genetics, so they would have found the focus on a relatively small range of species surprising. This aspect might have made some physiological concepts more difficult to approach. For example, competence to flower in response to day length has been difficult to explain mechanistically in Arabidopsis, because this species responds to photoperiod so strongly and rapidly after germination. Nevertheless, the relative ease of genomic sequencing and the availability of CRISPR-cas9 reverse genetics has begun to broaden the range of species whose flowering behavior can be addressed genetically (Zhai et al. 2024; Sashidhar and Coupland 2026). Also, some unifying phenomena have emerged among angiosperms, such as the photoperiodic induction of *FT* transcription in leaves to induce flowering, which is a common feature of SD and LD species in the dicotyledonous and monocotyledonous lineages, supporting the universality of florigen predicted from grafting studies. However, the diversity of mechanisms conferring day-length-dependent *FT* transcription could not have been predicted. Similarly, florigen was not expected by the physiologists to be a protein, and the gene regulatory networks activated by FT at the SAM and their cellular specificity are not yet sufficiently elaborated to discern whether common features exist, for example, between Arabidopsis and cereals, although some MADS-box transcription factor genes appear to be conserved (Tsuji et al. 2013). As mentioned above, early work highlighted the importance of sink–source relationships in floral transition, and the idea that the SAM becomes a stronger sink for photosynthate during the early stages of floral transition was considered critical. To date, there is little genetic understanding of sink strength and its contribution to flowering; however, this might influence the transport of FT to the SAM through the phloem. Moreover, intriguingly, in mutants for TREHALOSE 6 PHOSPHATE SYNTHASE 1 (TPS1)—the enzyme that catalyzes the synthesis of T6P—*FT* transcription is strongly reduced, and the *tps1* mutant is one of the few Arabidopsis mutants in which floral transition is completely prevented (Wahl et al. 2013). The biochemical mechanisms by which TPS1 is linked to flowering may involve regulation of the protein kinase SNF-RELATED KINASE 1 (SnRK1) (Van Leene et al. 2022), but whether this is linked to the original concepts of source–sink relationships remains unclear. Similarly, phytohormone levels and signaling were long thought to be key aspects of floral transition (Bernier 1988), but they remain only sketchily understood. In Arabidopsis, gibberellins are promoters of floral transition that appear also to inhibit aspects of floral development (Yamaguchi et al. 2014; Kinoshita et al. 2020), but much deeper understanding of the temporal–spatial patterns of gibberellin action is required. Similarly, cytokinins were long associated with changes in SAM morphology during floral transition, and although some genetic analyses and imaging in rice and Arabidopsis support a role (Sato et al. 2025; Bartrina et al. 2025), it remains to be fully mechanistically integrated into the flowering regulatory network. Furthermore, the nature of the temperature-sensing mechanism underlying the vernalization response has not yet been defined at the biochemical level. This might reside in the detailed biochemistry of higher-order regulatory complexes, for example in the details of the mechanisms of assembly of FRI condensates in cold, or in the assembly of specific protein complexes that modify histones on genes that control flowering time during vernalization. To date, no universal component of vernalization response has been identified, suggesting that this might have evolved independently in many angiosperm families. For example, a central role for repression of *FLC* transcription by vernalization seems specific to the Brassicaceae and not to be shared by legumes or cereals (Yan et al. 2004; Jaudal et al. 2013). Nevertheless, some commonality may exist at the regulatory level and in the cold-sensing mechanism, for example, and although the precise modifications differ, changes in histone modification on flowering genes during vernalization appear to be broadly shared (Yang et al. 2014; Liu et al. 2024). Identifying the common regulatory features of the vernalization response in different species might lead to a universal underlying cold-sensing mechanism. Overall, future studies of floral induction and its regulation by environmental cues promise to be as exciting as those of the past, and I wonder how the field will look at the bicentennial of *Planta*?

## Data Availability

This review article does not contain any new data.
